# Past aquatic environments in the Levant inferred from stable isotope compositions of carbonate and phosphate in fish teeth

**DOI:** 10.1371/journal.pone.0220390

**Published:** 2019-07-31

**Authors:** Guy Sisma-Ventura, Thomas Tütken, Stefan T. M. Peters, Or M. Bialik, Irit Zohar, Andreas Pack

**Affiliations:** 1 Israel Oceanographic & Limnological Research, Haifa, Israel; 2 Department of Isotope Geology, Georg-August-University of Göttingen, Göttingen, Gemany; 3 Institute of Geosciences, Johannes Gutenberg-University of Mainz, Mainz, Germany; 4 Department of Marine Geosciences, Charney School of Marine Sciences, University of Haifa, Haifa, Israel; 5 Oranim Academic College, Kiryat Tivon, Israel; Universite de Bourgogne, FRANCE

## Abstract

Here we explore the carbon and oxygen isotope compositions of the co-existing carbonate and phosphate fractions of fish tooth enameloid as a tool to reconstruct past aquatic fish environments and harvesting grounds. The enameloid oxygen isotope compositions of the phosphate fraction (δ^18^O_PO4_) vary by as much as ~4‰ for migratory marine fish such as gilthead seabream (*Sparus aurata*), predominantly reflecting the different saline habitats it occupies during its life cycle. The offset in enameloid Δ^18^O_CO3_-_PO4_ values of modern marine Sparidae and freshwater Cyprinidae from the Southeast Mediterranean region vary between 8.1 and 11.0‰, similar to values reported for modern sharks. The mean δ^13^C of modern adult *S*. *aurata* and *Cyprinus carpio* teeth of 0.1±0.4‰ and -6.1±0.7‰, respectively, mainly reflect the difference in δ^13^C of dissolved inorganic carbon (DIC) of the ambient water and dietary carbon sources. The enameloid Δ^18^O_CO3_-_PO4_ and δ^13^C values of ancient *S*. *aurata* (Holocene) and fossil *Luciobarbus* sp. (Cyprinidae; mid Pleistocene) teeth agree well with those of modern specimens, implying little diagenetic alteration of these tooth samples. Paired δ^18^O_PO4_-δ^13^C data from ancient *S*. *aurata* teeth indicate that hypersaline water bodies formed in the Levant region during the Late Holocene from typical Mediterranean coastal water with high evaporation rates and limited carbon input from terrestrial sources. Sparid tooth stable isotopes further suggest that coastal lagoons in the Eastern Mediterranean had already formed by the Early Holocene and were influenced by terrestrial carbon sources. Overall, combined enameloid oxygen and carbon isotope analysis of fish teeth is a powerful tool to infer the hydrologic evolution of aquatic environments and assess past fishing grounds of human populations in antiquity.

## Introduction

Fish live in nearly all aquatic environments worldwide, from mountain streams to the abyssal depths of the world’s oceans. Mineralised in isotopic equilibrium with body fluid, the phosphate oxygen isotope composition (δ^18^O_PO4_) of fish bioapatite is a function of both the temperature and the oxygen isotope composition (δ^18^O_Water_) of ambient water [1–4]. Bony as well as cartilaginous fish mineralize enameloid as crown-forming dental tissue. Enameloid consists of bioapatite that often has a high fluorine content, similar to that of fluorapatite[5–7], especially in sharks [8]. However, enameloid F-content in bony fish (teleosts) can vary between species and within species, depending to developmental stage [5, 9]. The teleost fish analysed in this study belong to species with high F-content (*Sparus* ~3%) as well as low F-content (*Carpio* ~0.3%) [9]. Fish tooth enameloid is highly resistant to diagenetic alterations following post-mortem burial [10]. Fish tooth δ^18^O_PO4_ values therefore serve as excellent proxies for ancient aquatic habitats (i.e. rivers, lakes, lagoons and oceans; e.g., [11–20]) and enable us to infer the salinity of past fishing grounds [21–22].

During biomineralisation of teeth some carbonate ions replace phosphate (Type-B) and OH-F sites (Type-A) in the crystal lattice of bioapatite (hydroxylapatite): i.e., Ca_5_(PO_4_, CO_3_)_3_(OH, F, CO_3_) [23]. The oxygen and carbon isotope composition (δ^18^O_CO3_ and δ^13^C values, respectively) of this structural carbonate can be used as a proxy to infer fish (palaeo-) environments (e.g.,[4, 19–20, 24–25]), by indicating different sources of carbon (marine vs. fresh water/terrestrial). However, compared to the phosphate fraction (δ^18^O_PO4_), the δ^18^O of the carbonate fraction of tooth enamel(oid) is more prone to diagenetic alteration [12, 25–27]. Moreover, δ^18^O_CO3_ values exhibit high, unsystematic variability up to 5–6‰ in modern shark teeth, even within tooth samples from the same individual, suggesting that δ^18^O_CO3_ in fish teeth might not be an ideal palaeo-environmental proxy [24]. However, δ^18^O_CO3_—which systematically co-varies with δ^18^O_PO4_ in mammal teeth [28]—is frequently used in conjunction with δ^18^O_PO4_ as a tool to assess the degree of diagenetic alteration in fossil tooth samples (e.g.,[29–30]).

Large, but systematic variations in δ^13^C values have also been observed between the dentine and the enameloid of modern shark teeth [24]. The positive δ^13^C values of enameloid (1.6 to 4.8‰) compared to significantly lower values in dentine (-6.4 to -2.3‰) suggest that enameloid approaches isotopic equilibrium with ambient dissolved inorganic carbon (DIC), likely through diffusional exchange with seawater DIC [24]. The δ^13^C values of structural carbonate of enameloid in fossil shark teeth agree well with those documented in modern species, suggesting that *in vivo* carbon isotope compositions are preserved [19–20, 25]. Thus, in cases where the original stable isotope compositions of fish teeth are preserved, the δ^13^C values of the enameloid may serve as a proxy for changes in the isotopic composition of DIC of the ambient water [19–20, 24–25]. Yet, the range of enameloid δ^13^C values in modern bony fish, which is essential to assess fossil fish δ^13^C values, is presently limited.

In this study, we use carbon and oxygen isotopic compositions of co-existing carbonate and phosphate fractions of fish tooth enameloid to infer the aquatic environments in which (pre-)historic humans caught their fish. For this purpose, different fish species from the modern Mediterranean (Sparidae) and freshwater fish from Lake Kinneret (Cyprinidae) were analyzed for their δ^18^O_CO3_, δ^18^O_PO4_ and δ^13^C_CO3_ values. These results are then compared to values obtained from fish teeth (belonging to the same species) recovered from different archaeological sites and periods in the southern Levant. To examine the possible effect of diagenesis on enameloid carbon and oxygen isotopic compositions, we also analysed some fossil pharyngeal teeth (1^st^ molariform tooth) recovered in deposits of lake ‘Ubediya (Lower palaeolithic, ca. 1.4 Ma; Jordan Rift Valley). Combined δ^18^O_PO4_-δ^13^C and δ^18^O_CO3_-δ^13^C data of fish tooth enameloid is explored as a tool to reconstruct the hydrological history of past fish habitats.

## Materials and methods

### Materials

Teeth of five extant fish species from three different aquatic habitats in the Levant were analysed for their enameloid carbon and oxygen isotope composition: *Sparus aurata* (*n* = 3, 9 teeth) and *Pagrus caeruleostictus* (*n* = 2, 6 teeth) (Sparidae, Perciformes, seabream) from the Mediterranean Sea (Haifa Bay, Israel), *Scarus* sp. (Scaridae; parrotfish, *n* = 1, 3 teeth) from the Red Sea, and the domesticated *Cyprinus carpio* (Cyprinidae; common carp, *n* = 2, 6 teeth) from the freshwater of Lake Kinneret in northern Israel (Table 1). Each of these species has a distinct diet: *S*. *aurata* feeds mainly on shellfish, mussels and oysters; *P*. *caeruleostictus* feeds on bivalves, crustaceans and fish; *Scarus* sp. feeds on corals and sponges; *C*. *carpio* feeds on plants, insects, crustaceans and benthic worms. Enameloid δ^18^O_CO3_, δ^18^O_PO4_ and δ^13^C_CO3_ values of these fish are used as reference data for comparison with those of ancient fish teeth of the same species.

**Table 1:** Stable isotope composition of enameloid of modern fish teeth (The Sparidae δ^18^O_PO4_ values were published in Sisma-Ventura et al. [17]).

Sample	Location	δ^18^O_PO4_	δ^13^C_CO3_	δ^18^O_CO3_	Δ^18^O_CO3-PO4_
Diet		[‰ VSMOW]	[‰ VPDB]	[‰ VSMOW]	
*S*. *aurata* [500 g]	Haifa Bay	22.5	-1.8	32.5	10.1
Gastropods, bivalves	Mediterranean Sea	22.6	-2.1	31.6	9.0
		22.6	-3.0	31.1	8.5
*S*. *aurata* [640 g]		23.3	-0.1	32.4	9.1
		23.2	0.6	33.5	10.3
		23.2	-0.5	34.2	11.0
*P*. *caeruleostictus* [900 g]		23.3	-0.7	32.4	9.1
Crustacea, Mollusca		23.2	0.5	33.2	10.0
		23.2	0.3	32.1	8.9
*S*. *aurata* [950 g]		23.2	0.6	33.5	10.3
		23.4	0.1	32.3	8.9
		23.4	-0.1	33.3	10.0
*P*. *caeruleostictus* [2,500 g]		23.3	0.3	32.4	9.1
		23.3	0.6	33.5	10.2
		23.4	0.0	33.4	10.0
*C*. *carpio* [930 g]	Lake Kinneret	21.0	-6.2	30.5	9.5
Detritus, insects and macrophytes	Freshwater	21.1	-6.7	29.4	8.3
*C*. *carpio* [970 g]		21.7	-6.2	30.5	8.9
		21.7	-5.2	30.8	9.1
*Scarus* sp.	Eilat, Red Sea	23.4	3.8	34.4	11.0
Algae from live corals and stones					

The archaeological samples used in this study are the first molariform teeth of *Sparus aurata* (*n* = 45) of the family Sparidae (Perciformes, seabream). These fish teeth were excavated from 12 well-dated archaeological sites in Israel [22], covering key periods of the Holocene: (1) the Pre-Pottery Neolithic sub-periods (A, B and C) dated to the Early Holocene (11,700–8,500 yr BP); (2) the Chalcolithic, (7,500–5,700 yr BP); (3) the Late Bronze Age, (3,500–3,200 yr BP); (4) the Iron Age (3,200 and 2,700 yr BP); and the Byzantine Age (1,680–1,360 yr BP) (Table 2). Additionally, five fossil pharyngeal teeth (1^st^ molariform) belonging to *Luciobarbus* sp. (Cyprinidae), recovered from 'Ubeidiya in the Jordan Rift Valley and dated to ca. 1.4-million-years ago [31] were analysed (**Table 3**).

**Table 2:** Stable isotope composition of enameloid from ancient *Sparus aurata* (Sparidae) first molariform teeth (The Sparidae δ^18^O_PO4_ values were published in Sisma-Ventura et al. [17]).

Site	Archaeological Period	δ^18^O_PO4_	δ^13^C_CO3_	δ^18^O_CO3_	Δ^18^O_CO3-PO4_
		[‰ VSMOW]	[‰VSMOW]	[‰ VPDB]	
**Hatoula**	PPNA (9,750–8,500 BCE)	23.4	-4.5	31.8	8.4
		23.1	-2.0	30.2	7.1
		21.4	-1.8	29.5	8.1
**Hatoula**	PPNB (8,500–7,000 BCE)	24.1	-2.7	33.0	8.9
		25.4	-0.6	32.2	6.8
		22.4	-2.4	30.8	8.4
**Ashkelon-Afridar**	PPNC (7,000–6,500 BCE)	22.8	-0.8	30.9	8.1
		26.4	6.1	36.5	10.1
		23.0	-2.8	30.0	7.0
		21.1	-1.2	31.4	10.3
		22.6	0.8	29.7	7.1
**Gilat**	Chalcolithic (5,500–3,900/3,700 BCE)	21.6	-3.1	31.9	10.3
		22.8	-4.5	31.7	8.9
**Lachish**	LB III (first half of 12^th^ c. BCE)	24.8	0.0	33.8	9.0
		23.5	-2.2	35.2	11.7
		24.7	-0.1	34.3	9.6
**Tel Rehov**	LB IIA (late 14^th^ c. BCE)	25.1	1.0	32.3	7.2
	Late IA IIA (late 10^th^/ 9^th^ c. BCE)	24.7	-1.3	35.4	10.7
**Timna**	LB III (first half of 12^th^ c. BCE)	25.4	3.5	34.8	9.3
		25.6	4.3	34.1	8.6
		24.3	0.1	31.4	7.1
		25.2	3.2	36.6	11.4
		25.1	3.6	36.1	11.1
**Ashkelon**	Early IA I (2^nd^ half of 12^th^ c. BCE)	25.5	1.3	35.1	9.6
		25.1	0.6	33.1	8.0
		26.1	-0.6	32.9	6.8
	IA I (12^th^ c. BCE)	26.1	0.6	34.0	7.9
	IA I (12^th^-early 10^th^ c. BCE)	25.4	0.2	33.9	8.5
		24.2	-1.1	33.7	9.5
		25.6	-0.2	33.6	8.0
**Tel Miqne**	IA I (11^th^/early 10^th^ c. BCE)	26.1	0.4	34.3	8.2
	IA II (10^th^/9^th^ c. BCE)	24.3	-1.7	33.1	8.8
**Jerusalem pool**	IA II (late 9^th^/early 8^th^ c. BCE)	24.8	-1.6	32.6	7.8
		25.1	1.2	33.8	8.7
		23.8	-1.3	31.5	7.7
		24.5	0.9	32.3	7.8
		25.4	-0.8	34.0	8.6
		24.3	1.3	34.0	9.7
		23.9	-2.1	31.2	7.3
**Tel Taninim**	Byzantine (4^th^-7^th^ c. CE)	22.7	-2.4	30.1	7.4
**Haluzsa**	Byzantine (6^th^ c. CE)	24.6	1.3	35.3	10.7
		25.3	0.3	33.1	7.8
**Shivta**		25.4	3.0	34.0	8.6
		25.6	1.1	33.4	7.8
**Tamra**	Early Islamic (early 8^th^ c, CE)	25.9	2.1	34.8	8.9

**Table 3:** Stable isotope composition of enameloid from fossil Cyprinidae teeth.

Site	Layer	δ^18^O_PO4_	δ^13^C_CO3_	δ^18^O_CO3_	Δ^18^O_CO3-PO4_
		[‰ VSMOW]	[‰ VPDB]	[‰VSMOW]	
'Ubeidiya	II38	19.22	-3.11	29.19	9.97
		20.23	-3.57	28.42	8.19
		20.65	-3.99	29.10	8.45
		19.64	-3.22	28.43	8.79
	II23	17.41	-6.39	25.51	8.10

The fossil fish were identified to species level using reference collections housed at the University of Haifa (Israel), the National Natural History Collections at the Hebrew University and The Zooarchaeological collections at the Autonomous University of Madrid (Spain).

### Oxygen and carbon isotope analyses of the carbonate fraction in bioapatite

The enameloid part (~0.2–0.4 mm layer) of each individual tooth crown was separated from the dentine using a diamond-head micro-dental drill, washed three times with distilled water and dried overnight at 50°C. Each sample was crushed and ground to powder using an agate mortar and pestle. Organic matter was removed from the sample powder by soaking in 30% H_2_O_2_ over night as described in Gehler et al. [32]. This pretreatment was tested on shark’s teeth (dentine + enameloid) and seemed to have minor effect on the δ^13^C values, which were comparable to those obtained using different pretreatments, such as NaOCl + acetic acid, as well as untreated samples. [24] The pretreatment effect on the δ^18^O_CO3_ values, observed in shark’s teeth [24] was minimised [26] by removing the dentine entirely from the enameloid. Due to low average CO_3_^2−^ content (2–3 wt %), approximately 2 mg of enameloid powder was reacted with 100% H_3_PO_4_ at 70°C in a Thermo Scientific KIEL IV automated carbonate device. The δ^18^O_CO3_ and δ^13^C values of the samples were then determined on the CO_2_ using a Finnigan Delta Plus gas source mass spectrometer in dual inlet mode at the stable isotope laboratory of the Geoscience Center at the University of Göttingen, Germany.

Measured isotope ratios were normalised to an in-house carbonate standard (Solnhofen) which has been calibrated against NBS-19. The analytical precision of this method is better than ±0.1‰ for both δ^18^O_CO3_ and δ^13^C. Measured isotope ratios are reported in δ-notation, i.e., as the deviation in per mil (‰) from the international measurement standards Vienna Standard Mean Ocean Water (VSMOW; δ^18^O_CO3_) and Vienna Pee Dee Belemnite (VPDB; δ^13^C):

$$\delta_{Sample}=[R_{Sample}/R_{Standard}-1]\times 10^{3}$$

where R represents the ^18^O/^16^O or ^13^C/^12^C ratio.

The analytical precision of the in-house carbonate standard was ±0.08‰ (1σ) in δ^18^O_CO3_ and ±0.05‰ (1σ) in δ^13^C (*n* = 16). For untreated samples of the NBS 120c Florida phosphate rock standard, we obtained a δ^18^O_CO3_ value of 30.1±0.12‰ and a δ^13^C value of -6.3±0.05‰ (*n* = 16). The reproducibility of the in-house bioapatite standard AG LOX (modern African elephant enamel; [27]) for untreated samples was 29.9±0.14‰ for δ^18^O_CO3_ and -11.75±0.04‰ for δ^13^C (*n* = 16). These mean values compare well with the values of 30.30±0.17‰ for δ^18^O_CO3_ and -11.78±0.12‰ (*n* = 23) for δ^13^C reported for untreated AG LOX by Wacker et al. [33].

### Oxygen isotope analyses of the phosphate fraction in bioapatite

The phosphate fraction of the samples was separated using a method modified after Dettmann et al. [34] and described in detail by Tütken et al. [35] and Sisma-Ventura et al. [22]. Five milligrams of pretreated enameloid powder were placed on vibrating table and digested in 0.8 ml HF (2 M) for 12 h. The samples were centrifuged, and the remaining supernatant solution with the dissolved phosphate was separated from the CaF_2_ precipitates. The HF solution transferred into a new vial and was neutralised with 25% NH_4_OH using bromothymol blue as pH indicator, and Ag_3_PO_4_ precipitation was completed by adding 0.8 ml of 2 M AgNO_3_. The settled Ag_3_PO_4_ crystals were centrifuged, and the supernatant solution containing excess AgNO_3_ was removed. The Ag_3_PO_4_ precipitate was then rinsed five times with Milli-Q water and dried overnight in an oven at 50°C.

Ag_3_PO_4_ aliquots of 0.5 mg were placed into silver capsules and analysed in triplicate by means of high temperature reduction using a Finnigan TC-EA coupled via a Conflo III to a Micromass 100 GC-IRMS at the University of Mainz, or by a Finnigan Delta Plus XL GC-IRMS at the Universities of Tübingen and Lausanne, according to the method of Vennemann et al. [36]. The raw δ^18^O_PO4_ values were normalised to an Ag_3_PO_4_ standard with a certified value of 21.7‰ (silver phosphate P/N IVA33802207, batch no. 180097, distributed by IVA Analysentechnik, Germany). The analytical precision for this standard was better than ±0.3‰ (1σ). For untreated NIST SRM 120c Florida phosphate rock standard reference material, we obtained a δ^18^O_PO4_ value of 21.9±0.3‰ (n = 15). This value compares well with the value of around 21.7‰ initially proposed by Lécuyer et al. [12] and measured in most other laboratories as compiled in Chenery et al. [37].

## Results

### Oxygen and carbon isotope compositions of carbonate and phosphate in modern and ancient fish tooth enameloid

Individual δ^18^O_CO3_, δ^18^O_PO4_ and δ^13^C data from the analyses of bulk enameloid powder from five modern Sparidae and two modern Cyprinidae are summarised in **Table 1**. The δ^18^O_CO3_ values of six teeth from two adult *S*. *aurata* (> 0.65–1.0 kg) are very similar and range between 32.3 and 34.2‰, while those of two adult *P*. *caeruleostictus* (> 0.9–2.5 kg, *n* = 6) range between 31.4 and 33.5‰. A similar δ^18^O_CO3_ range between 31.1 and 32.5‰, was obtained for three teeth of a single juvenile sample of *S*. *aurata* (<0.5 kg). The ancient Sparidae teeth yield variable δ^18^O_CO3_ values (*n* = 45), spanning a broad range between 29.5 and 36.6‰ (**Table 2**).

The modern samples of Sparidae from the southeast Mediterranean littoral yield a relatively narrow δ^18^O_PO4_ range of about 1.0‰ between 22.5 and 23.4‰ (*n* = 15). In contrast, δ^18^O_PO4_ values between 21.1 and 26.4‰ were measured for the ancient *S*. *aurata* teeth (*n* = 45).

A narrow range between -0.5 and 0.6‰ is also observed for the δ^13^C values of modern adult *S*. *aurata* specimens (**Table 1**). A lower δ^13^C value, averaging -2.3±0.6‰ was found for teeth of a single juvenile sample of *S*. *aurata* (<0.5 kg). For adult *P*. *caeruleostictus* the δ^13^C values range between -0.7 and 0.6‰. No significant differences are present between the average δ^13^C values of the adult *S*. *aurata* and *P*. *caeruleostictus* samples. The ancient Sparidae teeth (**Table 2**) yield variable δ^13^C values, covering a large range from -5.8 to 6.1‰.

Modern carp teeth enameloid yield relatively narrow ranges between 21.0 and 21.7‰ for δ^18^O_PO4_, between 29.4 and 30.8‰ for δ^18^O_CO3_, and between -5.2 and -6.6‰ for δ^13^C (*n* = 4; **Table 1**). Fossil carp enameloid values range between 16.8 and 20.7‰ for δ^18^O_PO4_, 25.5 and 29.2‰ for δ^18^O_CO3_, and -6.4 and -3.1‰ for δ^13^C (*n* = 5; **Table 3**).

### The theoretical δ^18^O_PO4_ range of Sparidae bioapatite

**Fig 1:**
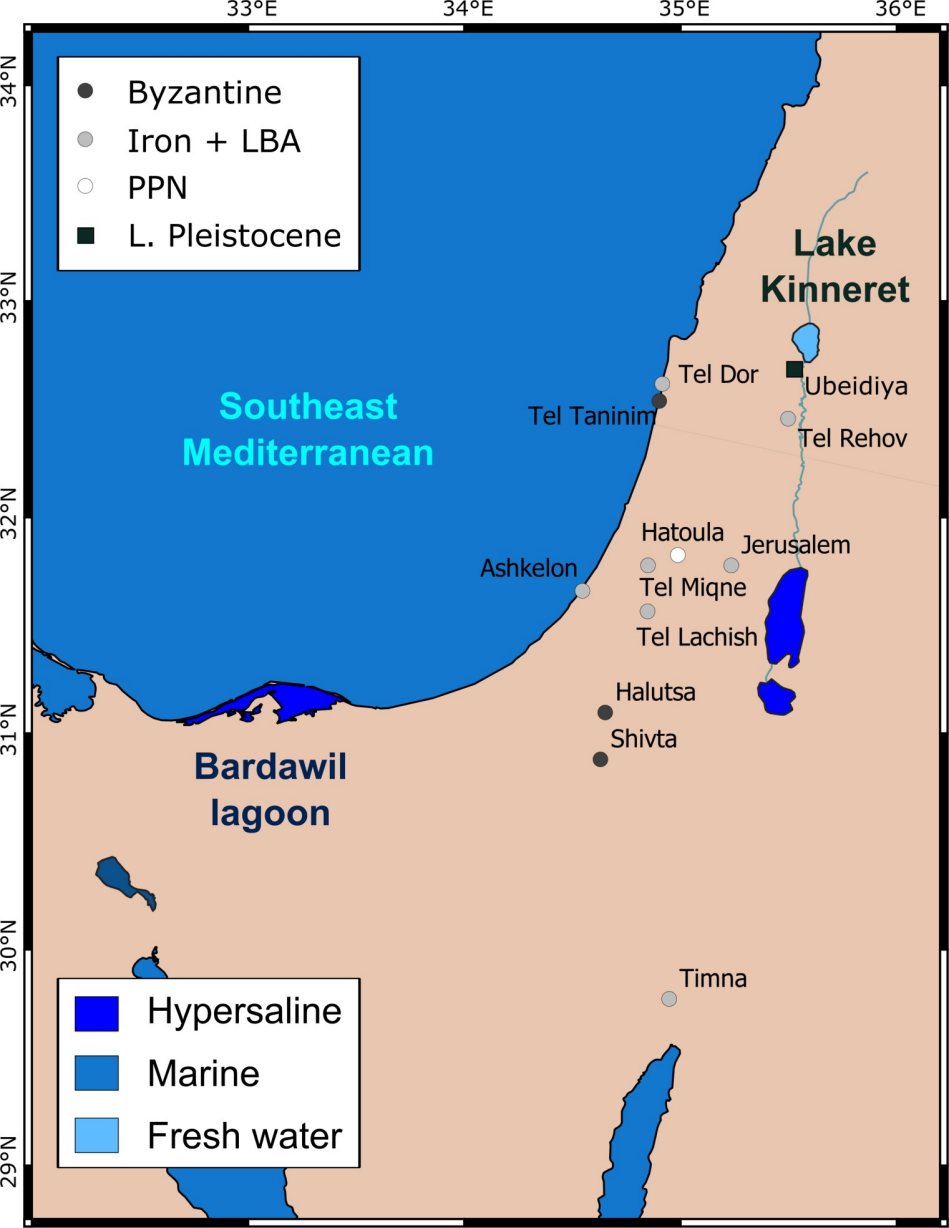
Study site map with archaeological sites from which the ancient *Sparus aurata* teeth were excavated with according archaeological periods: PPNA = Pre-pottery Neolithic, LBA = Late Bronze Age, Iron Age, Byzantine period, as well as fossil carp teeth from the Early Pleistocene palaeolake Ubeidiya. See text for exact age ranges of the periods.

The calculation of the equilibrium δ^18^O_PO4_ range of bioapatite forming in the southeast Mediterranean littoral and in hypersaline lagoons, evolving from typical seawater of this region, is based on the temperature-dependent relation for the oxygen isotope fractionation during biomineralisation of apatite by Lécuyer et al. [4]:

$$T\left({}^{\circ}\text{C}\right)=117.4-4.5\times (\delta^{18}O_{PO4}-\delta^{18}O_{SeaWater}$$

where δ^18^O_PO4_ and δ^18^O_SeaWater_ correspond to the isotope compositions of bioapatite and seawater relative to VSMOW, respectively. This relationship is valid for the temperature range of 8°C < T < 32°C. This equation from Lécuyer et al. [4] was established using bioapatite from both modern lingulids and sharks, yielding consistent temperatures from bioapatites equivalent to those from co-existing carbonates, therefore, providing the most reliable estimates of aquatic palaeotemperatures for bioapatites. Hence, we use the Lécuyer et al. [4] equation for calculating the equilibrium δ^18^O_PO4_ range of bioapatite. Likewise, the equation by Pucèat et al. [3] for the temperature-dependent phosphate oxygen isotope fractionation is widely used to calculate the equilibrium δ^18^O_PO4_ range of bioapatite. Surface water temperatures of the East Mediterranean range from 15°C in late winter (February-March) to 30°C in summer (July-August). The range of δ^18^O_SeaWater_ recorded from the East Mediterranean is relatively small [38–39], varying between 1.4‰ (February-March) and 1.8‰ (July-August). The calculated range of expected bioapatite δ^18^O_PO4_ forming in the southeast Mediterranean is slightly less than 3.0‰ and varies between 21.1 and 23.7‰. The δ^18^O_PO4_ values of modern Sparidae which were caught in the southeast Mediterranean littoral vary between 21.5 and 23.4‰, therefore agreeing well with the calculated range [21–22]. Significantly higher δ^18^O_PO4_ values, between 23.6 and 25.4‰ [2], were measured in teeth of fish from the hypersaline Bardawil lagoon of Northern Sinai, Egypt. The Bardawil lagoon (**Fig 1**) is a large (30 km long, 14 km max. width), shallow (0.3–3 m deep) hypersaline coastal lagoon, separated from the Mediterranean Sea by a narrow sandbar. The Bardawil is connected to the sea via two small natural inlets (*Boughaz Zaranik*). Water exchange in the lagoon is controlled by Mediterranean Sea tides with a mean height of 50 cm. As a result, it has an elevated salinity level and δ^18^O_Water_ values around 3.7‰ (range: 1.8‰ near the Mediterranean inlet, reflecting inflowing seawater, up to 7.2‰ due to high evaporation rates [2, 22]). Here we use the theoretical range of δ^18^O_PO4_ values expected for both ecosystems (southeast Mediterranean and the Bardawil lagoon) as endmembers to infer the palaeo-environments in which the fish were caught.

## Discussion

### Enameloid δ^18^O_PO4_ and δ^18^O_CO3_ of Sparidae and Cyprinidae teeth

The difference between the δ^18^O_CO3_ and the δ^18^O_PO4_ in bioapatite of about +9‰ is in agreement with the theoretical predictions of equilibrium precipitation of the phosphate and the carbonate fractions simultaneously from the same body fluids according to the phosphate-water and carbonate-water temperature equations [1, 4, 30]. However, the difference between δ^18^O_CO3_ and δ^18^O_PO4_ (i.e., Δ^18^O_CO3_-_PO4_) of tooth enamel can vary between species and sometimes also within a single tooth ([30] and refs. therein). For example, Δ^18^O_CO3_-_PO4_ in tooth enamel of modern mammals ranges between 7.2 and 10.4‰ (e.g., [30, 40]) and can even reach a variation of up to 2.2‰ within the same tooth ([30], and refs. therein). Similarly high variability was also observed for Δ^18^O_CO3_-_PO4_ in modern shark teeth (enameloid and dentine), which were found to vary between 6.9 and 11.8‰ [24]. The intra jaw inter-tooth Δ^18^O_CO3_-_PO4_ variation in a single modern shark can also vary by more than 2‰ [24].

**Fig 2:**
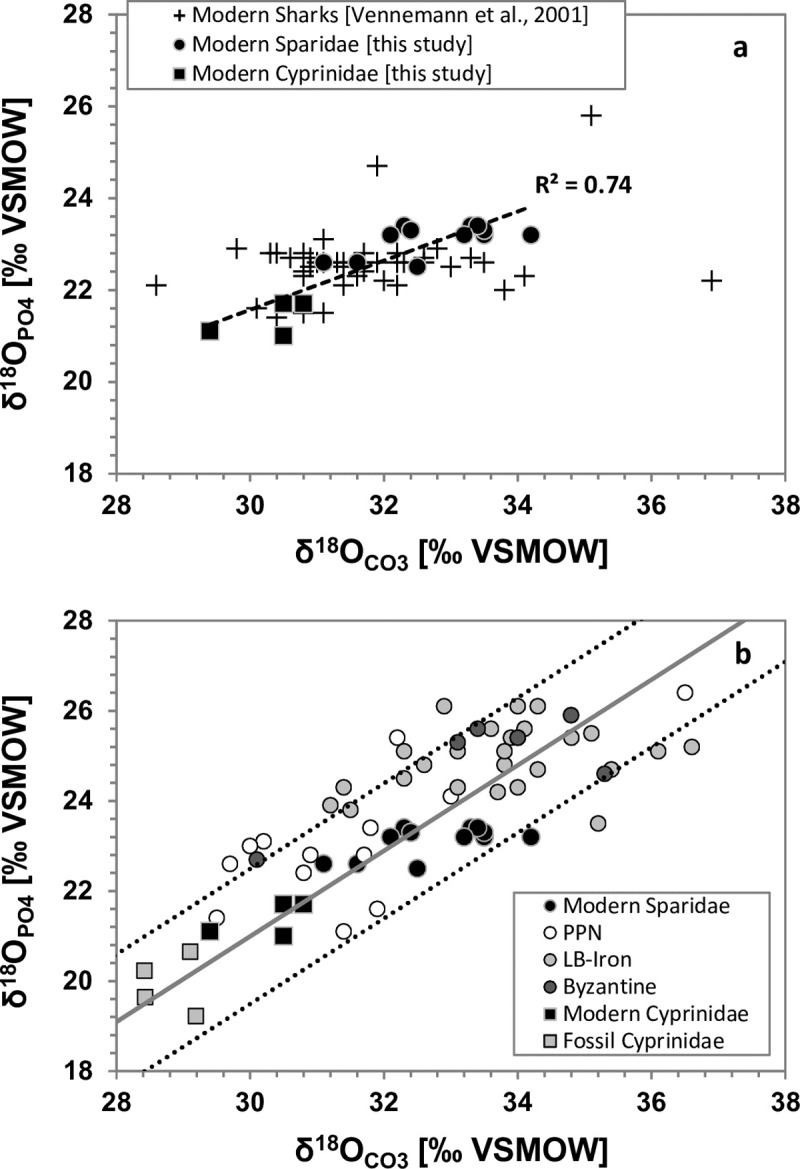
The difference between δ^18^O of co-existing carbonate and phosphate fractions in enameloid of modern and ancient Sparidae and Cyprinidae teeth. (a) modern sharks, Sparidae and Cyprinidae (b) modern and ancient Sparidae as well as Cyprinidae. The black bold trend line: represents the δ^18^O_PO4_/δ^18^O_CO3_ relationship of modern mammals after Pellegrini et al. [25]. Note, the Δ^18^O_CO3_-_PO4_ prediction interval (PI) uncertainty of ±1.5‰ (stippled line) reported by Pellegrini et al. [25] agrees well with the range of modern shark teeth [19] and all bony fish data of Sparidae and Cyprinidae fall in this range too. The modern δ^18^O_PO4_/δ^18^O_CO3_ relationship of Sparidae and Cyprinidae, although yielding high correlation, covers a narrow δ^18^O_PO4_/δ^18^O_CO3_ range to allow comparison with the δ^18^O_PO4_/δ^18^O_CO3_ relationship of mammals. More data is needed to establish the δ^18^O_PO4_/δ^18^O_CO3_ relationship in modern bony fish.

The Δ^18^O_CO3_-_PO4_ values of modern Sparidae and Cyprinidae enameloid samples vary between 8.1 and 11.0‰, yielding an average of 9.5±0.7‰, (*n* = 8 modern fish; **Table 1**). These values agree well with the average Δ^18^O_CO3_-_PO4_ of 9.1±1.5‰ (*n* = 12) reported for modern shark teeth [24]. Moreover, similar to sharks, high unsystematic variability (~3‰) was observed in the δ^18^O_CO3_ values of modern Sparidae teeth, while δ^18^O_PO4_ values vary less by <1‰ in the same teeth (**Fig 2A**). The high correlation between the δ^18^O_CO3_ and the δ^18^O_PO4_ in modern teeth (r^2^ = 0.73), indicates that both fractions precipitate simultaneously from the same body water pool [25]. However, the more variable δ^18^O_CO3_ values in modern bony fish and shark teeth in comparison to that of the δ^18^O_PO4_ (**Fig 2A**) suggests that the two fractions could be subjected to different exchange rates, thus integrating different ranges of ambient conditions. Further support for this distribution can be seen in the data obtained from ancient *S*. *aurata* teeth, particularly from the LB-Ir age. The larger scatter of the data obtained from the carbonate fraction of the ancient *S*. *aurata* teeth may, therefore, still reflect the in vivo distribution of δ^18^O_CO3_ values [19–20, 24], rather than the effect of diagenetic alteration.

The Δ^18^O_CO3_-_PO4_ values of ancient *S*. *aurata* teeth (ranging between 6.8 and 11.7‰, **Fig 2B**) and fossil Cyprinidae teeth (ranging between 8.2 and 10.0‰) are, in most cases, in good agreement with expected values for Δ^18^O_CO3_-_PO4_ in modern sharks [24] (**Fig 2A**). Furthermore, they are similar to Δ^18^O_CO3_-_PO4_ of mammalian bioapatite, for which diagenetic alteration of oxygen isotope composition is better quantified [28–30]. The 1.4-million-year-old fossil teeth of *Luciobarbus* sp. (Cyprinidae) from the palaeolake of 'Ubeidiya are also well preserved as they fall in the expected δ^18^O_PO4_-δ^18^O_CO3_ range for modern bioapatite (**Table 3**). Thus, the Δ^18^O_CO3_-_PO4_ offset is similar for enamel (biogenic hydroxylapatite) and enameloid (biogenic fluorapatite).

We calculated the offset between the predicted and measured Δ^18^O_CO3_-_PO4_ values using the δ^18^O_CO3_ to δ^18^O_PO4_ relationship by Pellegrini et al. [30] which is based on all available modern mammal data. The calculated offset for ancient *S*. *aurata* (1.0±0.7‰) and fossil *Luciobarbus* sp. (Cyprinidae) (0.6±0.36‰) from the Δ^18^O_CO3_-_PO4_ regression falls within the predicted interval of uncertainty for the δ^18^O_CO3_-δ^18^O_PO4_ regression for mammals (**Fig 2B**). This suggests that the majority of enameloid δ^18^O_CO3_ (and by implication δ^13^C) values of ancient *S*. *aurata* teeth and fossil Cyprinidae teeth are well preserved and likely were not affected by any significant diagenetic alteration.

### Enameloid δ^13^C of Sparidae teeth

δ^13^C values in animals vary as a function of environment and biochemical pathways. The largest difference in δ^13^C occurs between organic (e.g., lipids, carbohydrates, proteins, and collagen) and inorganic (e.g., shell carbonate or carbonate within bioapatite) carbon components (e.g., [41–43]). The δ^13^C values of structurally bound carbonate in the bioapatite of the bones and teeth of herbivorous mammals are ~6–15‰ higher than the dietary sources of these animals (e.g., [43–45]). With similar carbon isotope fractionation between tooth enamel bioapatite, breath CO_2_, and diet, the inter-species differences in the δ^13^C values of structurally bound carbonate in the bioapatite of mammals result primarily from differences in digestive physiology [44–45]. While the δ^13^C of dentine in shark teeth derives predominantly from a dietary carbon source, the enameloid of the same teeth is typically characterised by 6–8‰ higher δ^13^C values [24]. Secondary, *in vivo* diffusional processes and isotopic exchange with ambient water DIC during tooth formation have been suggested as possible explanations for the elevated δ^13^C values of shark tooth enameloid [24].

The carbon intake of adult Sparidae is mainly derived from their diet; food items include the soft tissues of bivalves, gastropods and crustaceans [46]. Changing dietary carbon sources at different ontogenetic stages of the fish life cycle may explain the somewhat lower mean δ^13^C value of -2.3‰ found in a single juvenile fish (< 0.5 kg). Indeed, wild *S*. *aurata* have been observed to forage for shellfish and zooplankton [47] in a manner that varies with the size of the fish [48]. For instance, soft-bodied animals such as polychaetes and small crustaceans predominantly occur in the stomach contents of small *S*. *aurata* (5–9 cm in length), while larger, hard-shelled prey such as barnacles, bivalves and other teleost fish are consumed by larger *S*. *aurata* (10 to > 25 cm in length) [48].

Typical fractionation between carbonate and dissolved carbon in the form of bicarbonate ions is 2.5‰ at 20°C [49]. Hence, for typical Mediterranean seawater DIC δ^13^C values of 1.0±0.3‰ [38–39] carbonate δ^13^C values of up to 3.5‰ would approach isotopic equilibrium with DIC. Such positive δ^13^C values between 1.6 and 4.8‰ have been recorded in the carbonate fraction of enameloid bioapatite of modern and ancient shark teeth [19–20, 24]. A similar value was also measured in this study in a single sample of *Scarus* sp. (Scaridea, δ^13^C = 3.8‰) from the Red Sea (raised in a seawater aquarium-open system). Structurally bound carbonate δ^13^C values in enameloid bioapatite of modern Sparidae samples did not reach such high values. Thus, their body water bicarbonate pool seems still to be influenced to some degree by their dietary carbon sources, while approaching the isotopic composition of DIC in their habitat.

**Fig 3:**
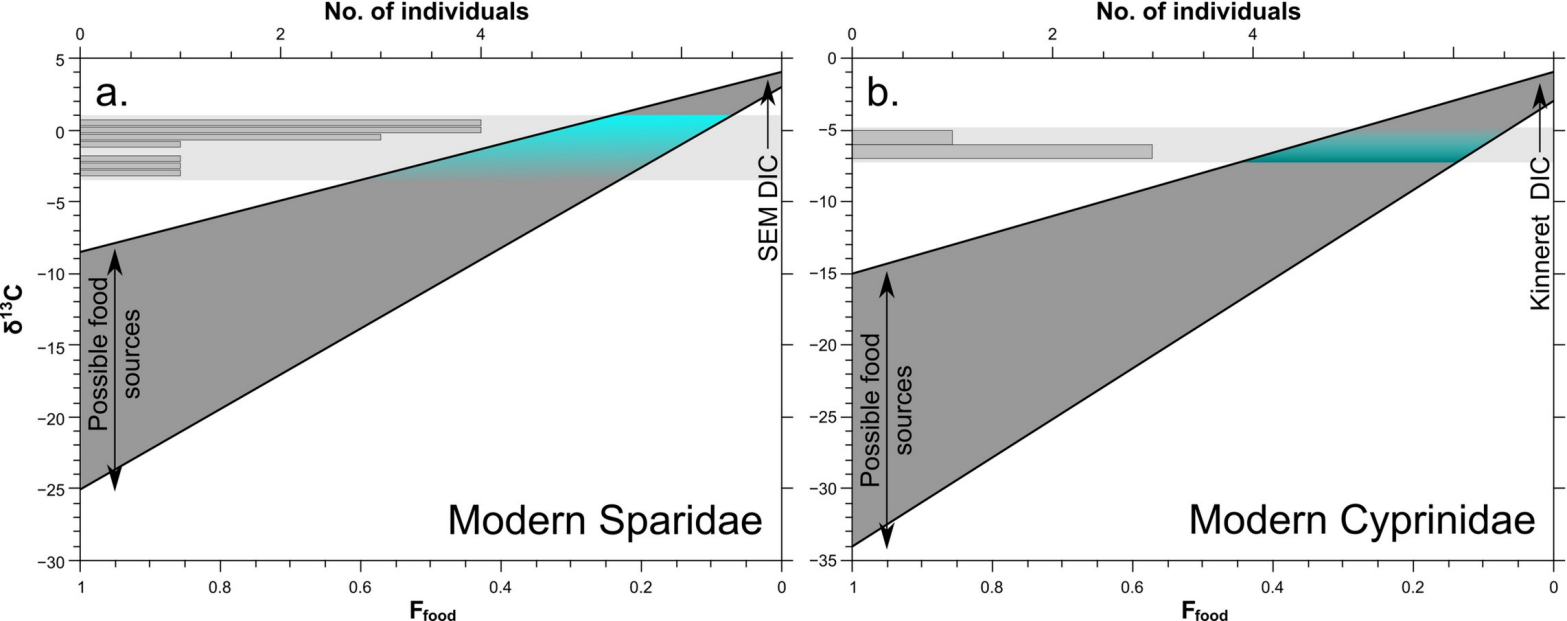
Estimated fraction of metabolic carbon from the diet (*f*_food_) in the carbonate δ^13^C values of modern Sparidae and Cyprinidae tooth enameloid from mixing calculation. For the δ^13^C_diet_ values of the food sources see text for details. Theoretical equilibrium δ^13^C_CO3_ of 3.5‰ and -2‰ for carbonate in fish enameloid for the South East Mediterranean (SEM) and Lake Kinneret, respectively, were calculated for an equilibrium fractionation of 2.5‰ (20°C), based on the δ^13^C_DIC_ of 1‰ and -5‰, respectively, for the two different water bodies [33–34, 48].

We estimate the percentage of metabolic carbon (*f*_food_) in the carbonate δ^13^C values of modern Sparidae and Cyprinidae teeth (**Fig 3**) by using a simple mixing calculation:

$$\left(\delta_{DIC}\times f_{DIC}\right)+\left(\delta_{Food}\times f_{Food}\right)+\alpha =\delta_{Enameloid}$$

$$f_{DIC}+f_{Food}=1$$

where δ^13^C of possible food sources for Sparidae range between -8.5 and -23‰ [50–51] and for Cyprinidae between -15 and -34‰ [52]. The δ^13^C of Mediterranean DIC is 1.0±0.3‰ [38–39] and between -4.0 and -7.0‰ for Lake Kinneret [53]. The *f*_DIC_ and the *f*_Food_ are the percent contribution of DIC and food, respectively, to the enameloid δ^13^C value (δ_enameloid_), and α is the fractionation factor between the dissolved carbon sources and the δ_enameloid_ value (assuming a 2.5‰ fractionation). The *f*_food_ in the δ^13^C of juvenile Sparidae range between 20% and 50%, while for adult fish (both Sparidae and Cyprinidae) it ranges between 10% and 30% (**Fig 3**). Assuming that the structural carbonate in the enameloid of bony fish has a similar carbon isotope fractionation as calcite (assuming 1.0‰ fractionation factor), and not aragonite (as assumed for sharks [24]), then the metabolic effect of both Sparidae and Cyprinidae would be much lower than estimated and their δ^13^C values would further approach isotopic equilibrium with the water DIC. The Scaridea δ^13^C value seems to approach isotopic equilibrium with the Red Sea DIC, but may also relate to its durophageous diet feeding on corals and sponges.

The δ^13^C of the freshwater Cyprinidae enameloid samples range between -6.6 and -5.2‰ while the lake water δ^13^C_DIC_ values vary between -7.0 and -4.0‰ (mean δ^13^C_DIC_ of around -5.5‰), with the Jordan River input ranging from -7.3 to -6.5‰ [53]. Thus, carbonate δ^13^C values of up to -2.0‰ would appear to approach isotopic equilibrium with the lake water DIC. Similar to Sparidae, Cyprinidae samples did not reach such high values, supporting the hypothesis that bioapatite δ^13^C values represent a mixture of ambient water δ^13^C_DIC_ and δ^13^C_diet_.

The main control on the δ^13^C_DIC_ in coastal lagoons is the mixing of fresh and marine waters entering the lagoon; meaning it is also a useful palaeosalinity indicator [54]. This is reflected in the co-variation of δ^18^O_PO4_-δ^13^C and δ^18^O_CO3_-δ^13^C values in tooth enameloid of *S*. *aurata*, which migrate between water bodies of different salinities [46–48]. Thus, variations in the δ^18^O_H2O_ and δ^13^C_DIC_ values of the lagoonal water are expected to be recorded in the bioapatite of the *S*. *aurata* teeth.

### Identifying the environmental history of fish ecosystems from paired δ^18^O_PO4-CO3_ and δ^13^C values

**Fig 4:**
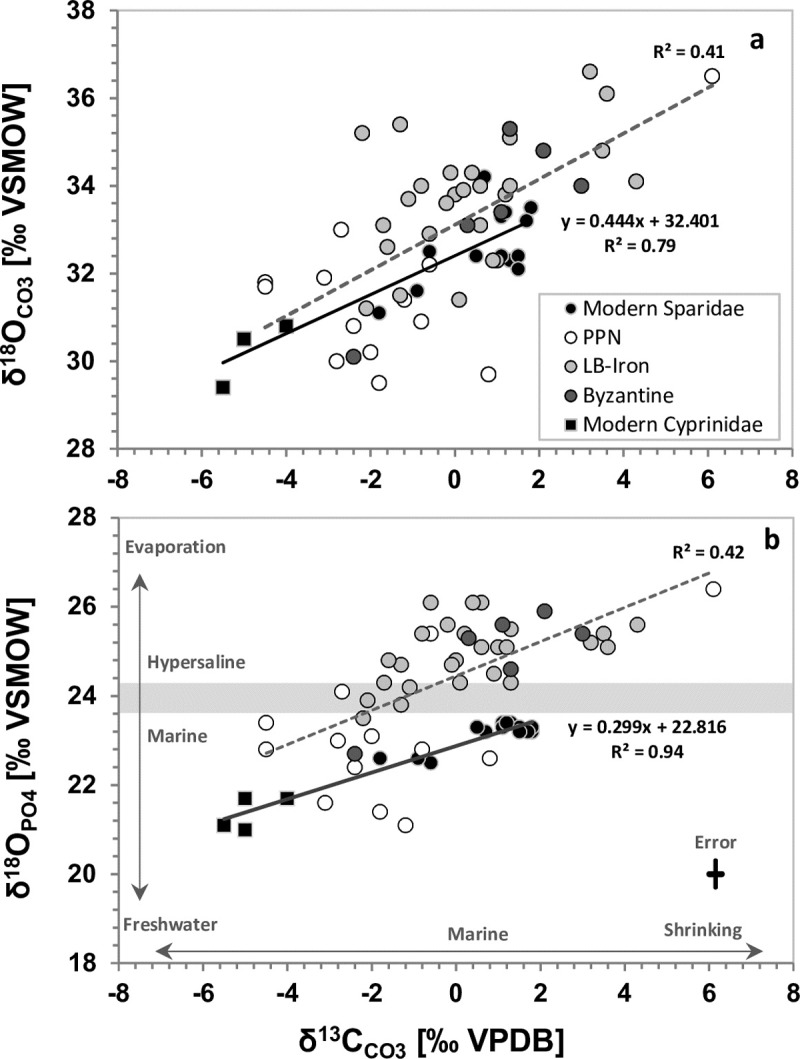
Covariance of (**a**) δ^18^O_CO3_-δ^13^C and (**b**) δ^18^O_PO4_-δ^13^C values of ancient and modern Sparidae as well as Cyprinidae tooth enameloid. The modern fish δ^13^C data is corrected for the Suess effect of -1.2‰ [33, 50] to pre-industrial values to enable comparison with the ancient Sparidae and Cyprenidae of the Southeast-Mediterranean region. Note that increasing δ^18^O values often also coincide with higher δ^13^C values recording evaporative changes in salinity and carbon source of the lagoonal water bodies these fish were caught from. A freshwater habitat (Lake Kinneret) of modern Cyprinidae is reflected primarily by the low δ^13^C, but also by δ^18^O values lower than those of marine Sparidae. Note that already in PPN some lagoons were mostly brackish-marine as indicated by low δ^13^C and δ^18^O values of the *Sparus* teeth but a few high values also imply the existence of hypersaline lagoons.

**Fig 4A and 4B** show paired δ^18^O_PO4_-δ^13^C and δ^18^O_CO3_-δ^13^C values from ancient *S*. *aurata* and fossil *Luciobarbus* sp. (Cyprinidae) teeth from the Levant, compared with the values of modern fish from the southeast Mediterranean coastal water and from Lake Kinneret, respectively. For comparison with pre-industrial values, modern fish tooth δ^13^C values were adjusted to the east Mediterranean Suess effect of ~1.2‰ on seawater δ^13^C_DIC_ (the depletion trend in oceanic δ^13^C_DIC_, due to the emission of CO_2_ from burning fossil fuels) obtained from the vermetid reef carbonate δ^13^C composite record [38, 55]. The ancient *S*. *aurata* teeth that bear the δ^18^O_PO4_ signature typical for bioapatite formed in Mediterranean coastal water [21–22] also fit the pre-industrial δ^13^C range for modern fish from this habitat. These values are mainly observed in the Early Holocene specimens, suggesting that the fish were caught in typical coastal water [22]. However, teeth with typical hypersaline δ^18^O_PO4_ values (> 23.7‰, [21–22] similar to those of extant fish from the Bardawil lagoon [2], show typical marine δ^13^C values (**Fig 4B**).

The highest δ^13^C values in the ancient *S*. *aurata* teeth are observed during the Mid-Late Holocene (Bronze Age and Iron Age) and are consistent with high δ^18^O_PO4_ values typical for tooth formation in hypersaline lagoons [2, 21–22]. The increase in the δ^13^C_DIC_ of closed, evaporative and warm water bodies, like the Bardawil lagoon, may be related to degassing of CO_2_ and seasonal biological effects, resulting in the high correlation between δ^18^O_PO4_ and δ^13^C in modern fish teeth (**Fig 4B**). Both, photosynthesis and degassing remove ^12^C-rich CO_2_ from the DIC pool of seawater leading to higher δ^13^C_DIC_ values ([56] and refs. therein). These effects are amplified when they occur in a limited isotopic reservoir (i.e. a restricted and shallow water bodies), such as a long-term lakes or lagoons [57–58]. Thus, similar to the coupling of δ^18^O_CO3_ and δ^13^C in carbonates of closed water bodies [57], the positive relationship between δ^18^O_PO4_-δ^13^C (r^2^ = 0.42; p<0.001) and δ^18^O_CO3_-δ^13^C (r^2^ = 0.41; p<0.001) values in the bioapatite of ancient and modern Sparidae teeth can be used as proxies for the formation of coastal lagoons.

The δ^18^O_PO4_ and δ^13^C values of the ancient fish teeth indicate that the hypersaline habitats of *S*. *aurata* in the Levant region formed during the Late Holocene from typical southeast Mediterranean coastal water under high evaporation rates and a limited carbon reservoir, similar to the present day Bardawil lagoon in Northern Sinai. This lagoon represents the main hypersaline habitat of Sparidae in the Levantine basin today [59] as well as in the past millennia [21–22]. These evaporatively-influenced lagoonal habitats evolved during the Mid-to-Late Holocene transition (~3.5 ka ago), when sea level stabilised close to its present-day level [60] approximately 3,620±160 years BP [61]. Sea-level stabilisation resulted in the formation of the perennial shallow hypersaline Bardawil lagoon along the northern Sinai coast, due to the establishment of long-shore currents that transported Nile sands which built up blocking sandbars [58, 62].

**Fig 5:**
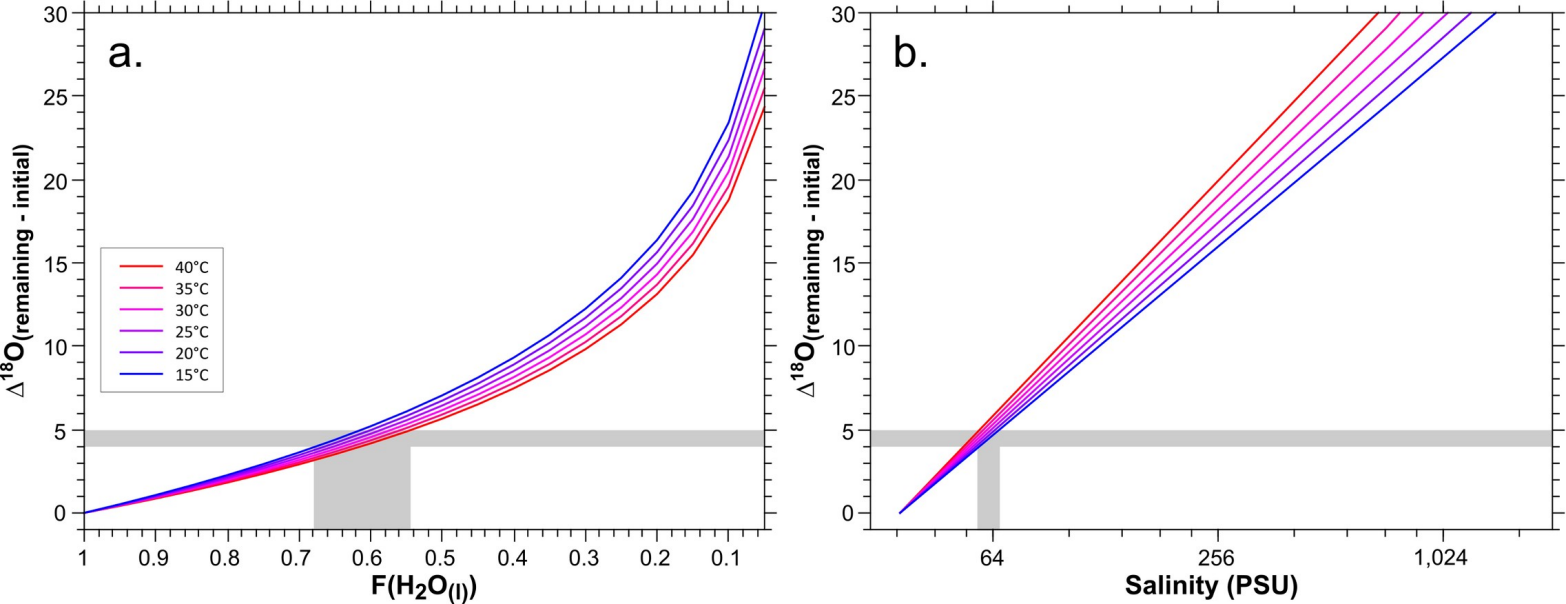
Calculated change in δ^18^O_water_ for different water temperatures (15–40°C) relative to initial conditions at 37‰ (PSU) for Mediterranean seawater as a function of (**a**) fraction of the initial water mass lost to vapor and (**b**) as a function of salinity. Horizontal bar illustrates the maximum range of δ^18^O_PO4_ difference observed in the Sparidae dataset, assuming this reflects only δ^18^O_H2O_ (i.e. salinity) differences in the ambient water; vertical grey bars represent the water loss and salinity, respectively, inferred from this maximum *Sparus* δ^18^O_PO4_ range for different water temperatures.

During evaporation, the lighter ^16^O isotope is preferentially fractionated into the gas phase, this results in the gas phase having ~9‰ (at 25°C, the mean annual surface temperature of water bodies in the study area) lower δ^18^O values then the water mass from which it evaporated [63]. This, for the most part, is an inreversable reaction. As such, the residual water mass becomes more fractionated in a way that could be described through Rayleigh distillation equations [64]. As such, if we know the initial δ^18^O value of the water mass, and how fractionated it became, we can calculate the loss of water to the vapor phase. As the salt does not evaporate, the residual water mass will retain the original amount of salt. Dividing the initial amount ot salt by the final mass of water will allow us to calculate the salinity. Taking the observed 4 to 5‰ difference in δ^18^O values between the modern (representing the non-evaporated water mass) and Late Holocene *S*. *aurata* tooth assemblages, it is thus possible to calculate the fraction of water lost (**Fig 5A**). Within a reasonable ambient temperature range for the SE Mediterranean region (15 to 30°C), this translates to a 33% to 46% loss of the initial water mass–indicating significant evaporation and restricted water exchange between lagoons and the open Mediterranean Sea during the Late Holocene. Assuming an initial seawater salinity of ~37‰, this loss would place the salinity in these basins to between 54‰ and 68‰ (**Fig 5B**), representing the upper range of habitable conditions for *S*. *aurata* [2, 59]. This is a maximum estimate, given that about 2.5‰ (of the 5‰ range) in *Sparus* teeth δ^18^O_PO4_ of the Bardawil lagoon can potentially be attributed to seasonal changes in water temperatures [22].

Our stable isotope results of *S*. *aurata* tooth enameloid indicate that an early phase of lagoon formation in the Levant was already taking place during the Pre-Pottery Neolithic period (**Fig 4**) between 11,000 and 6,000 yr BC (Early-mid Holocene, Greenlandian to mid Northgrippian), despite the lack of sedimentological evidence for such lagoons. However, δ^13^C values suggest that these lagoons were likely also fed by terrestrial sources. In fact, *Sparus* can tolerate a wide salinity range, including brackish water [46]. While most of the δ^18^O_PO4_ values of the PPN (Early Holocene) fish fall within the expected range of bioapatite formed in isotopic equilibrium with Mediterranean temperature and seawater δ^18^O values, some of the PPN samples have relatively low δ^13^C and δ^18^O_PO4_ values. These low δ^13^C and δ^18^O_PO4_ values fall along the regression between modern fish from the Mediterranean and Lake Kinneret (**Fig 4B**). This implies a freshwater influx (with low δ^13^C_DIC_ values) into past brackish habitats of *Sparus* during the PPN, Early Holocene. This provides evidence that *S*. *aurata* were caught in different lagoonal settings between the Early and Late Holocene.

Overall, the pairing of δ^18^O_PO4_-δ^13^C and δ^18^O_CO3_-δ^13^C in fish tooth enameloid thus represent a potential tool for investigating the hydrological history of marine to freshwater lagoonal settings and assessing the environments of past fish habitats. This isotopic approach can provide valuable information about past human fish exploitation patterns and enable us to distinguish marine from lagoonal fishing grounds.

## Conclusions

The environmental history of fish ecosystems in the southeast Mediterranean during the Early to Late Holocene was studied by investigating the stable isotope composition of Sparidae tooth enameloid. The provenance of Sparidae from marine versus hypersaline lagoonal water was recorded in the δ^18^O_PO4_, δ^18^O_CO3_ and δ^13^C values of modern and ancient fish teeth, which reflects the wide range of saline habitats encountered during their migratory life cycle. The enameloid δ^18^O_CO3_ as well as the Δδ^18^O_CO3-PO4_ offset and δ^13^C values of Holocene Sparidae teeth from the Levant are consistent with those of modern fish, indicating only minor diagenetic alteration. The δ^13^C of Sparidae tooth enameloid reflects the water DIC of the fish habitat and its diet. Thus, carbon cycling in past fish environments can be characterised using the δ^13^C of fish teeth.

Paired δ^18^O_PO4_-δ^13^C and δ^18^O_CO3_-δ^13^C values of ancient Sparidae tooth enameloid were used to infer the environmental history of fish from coastal and lagoonal settings in the Levant. Our results indicate that Late Holocene hypersaline lagoons evolved from typical southeast Mediterranean coastal water under high rates of evaporation and limited carbon input from terrestrial sources. This lagoon formation occurred in association with the postglacial stabilisation of the sea level about 3,500 years ago, however, first lagoons had already formed during the Early Holocene and were likely influenced by freshwater input. Overall, the pairing of δ^18^O_PO4_-δ^13^C and δ^18^O_CO3_-δ^13^C of fish tooth enameloid is therefore, a potential tool to infer past hydrological balance and environmental shifts of fish ecosystems. This enables us to gain new insights into past fishing grounds that were exploited by (pre-)historic humans.
